# The Metabolic Consequences of Pathogenic Variant in *FXYD2* Gene Encoding the Gamma Subunit of Sodium/Potassium-Transporting ATPase in Two Siblings with Sodium-Dependent Defect of Fructose, Galactose and Glucose Renal Reabsorption

**DOI:** 10.3390/genes16050535

**Published:** 2025-04-29

**Authors:** Jan Zawadzki, Ryszard Grenda, Agnieszka Madej-Pilarczyk, Elżbieta Ciara

**Affiliations:** 1 Department of Nephrology, Kidney Transplantation and Hypertension, The Children’s Memorial Health Institute, 04-730 Warsaw, Poland; jazaw4@wp.pl (J.Z.); adnerg@gmail.com (R.G.); 2 Department of Medical Genetics, The Children’s Memorial Health Institute, 04-730 Warsaw, Poland; a.madej-pilarczyk@ipczd.pl

**Keywords:** *FXYD2* gene, gamma subunit of Na,K-ATPase, fructose, galactose and glucose renal reabsorption, tubulopathy, endothelin-1 overproduction

## Abstract

**Background:** Abnormal sodium-dependent hexose reabsorption in the proximal tubule, accompanied by a functional decrease in sodium and water reabsorption under conditions of increased volemia, may be attributed to a dysfunction of primary transporters related to a genetic defect in the Na,K-ATPase gamma subunit. **Methods:** We examined two sisters, aged 6 and 8 years, who presented with hypercalciuria, glucosuria, fructosuria, galactosuria, and atypical proteinuria. Primary diabetes, galactosemia, and fructosemia were excluded, suggesting a defect in cellular hexose transport in the proximal tubule. We conducted tests on the family members to assess the impact of gradually increasing volemia, using a water-loading test, on tubular H^+^ transport and urinary excretion of calcium, citrate, endothelin-1 (ET-1), and atypical proteins. Whole-exome sequencing was performed in the affected patients to identify the genetic basis of this phenotype. **Results:** Extended investigations revealed a complex defect in tubular H^+^ transport, calcium and citrate handling, and atypical proteinuria, resulting from water load-driven overproduction of endothelin-1 (ET-1). Genetic analysis identified a heterozygous pathogenic variant, c.80G>A, p.(Arg27His), in the *FXYD2* gene, which encodes the gamma subunit of sodium/potassium-transporting ATPase. **Conclusions:** Our findings provide evidence that a defect in *FXYD2* (splice form a) leads to functional impairment of proximal tubular hexose reabsorption. This is the first report on the metabolic consequences of a pathogenic *FXYD2* variant affecting the gamma subunit of sodium/potassium-transporting ATPase in humans. The genotype–phenotype correlation in two siblings with a sodium-dependent defect in fructose, galactose, and glucose renal reabsorption allowed us to characterize a disease with a distinct clinical course and biochemical profile, not previously reported in the medical literature or genetic databases. Analysis of this condition was crucial for the early introduction of reno-protective treatment aimed at slowing the progression of nephropathy and for risk assessment in family members, which was essential for genetic counseling.

## 1. Introduction

Glucose and other sugars are the major energy source for human metabolic processes. Due to their polar structure, hexoses cannot cross cellular membranes by direct diffusion. Hexoses transport in the kidney primarily involves the reabsorption of glucose, fructose, and galactose through specialized transport mechanisms. The proximal convoluted tubule (PCT) is the specific segment of the nephron responsible for the majority of this transport. The PCT is the primary site for reabsorption of glucose and galactose via sodium-dependent transport (SGLT proteins) and fructose via facilitated diffusion (GLUT proteins). The PCT is divided into segments (initial—S1, middle—S2, final—S3), each with distinct roles in hexose transport. Glucose reabsorption occurs primarily through SGLT2, which is highly expressed in the S1/S2 segments. Galactose is reabsorbed via SGLT1, which is more prominent in the S3 segment. Their transport mechanisms effectively utilize the sodium gradient to reabsorb glucose or galactose from the tubular fluid. As sodium ions are reabsorbed, sugar is transported against its concentration gradient, relying on the sodium gradient established by the Na,K-ATPase pump [1]. The basolateral active extrusion of sodium by renal Na,K-ATPase enables the passive apical entry of sodium into the cells, often coupled with other solutes through various sodium-dependent transport systems, including co-transporters, channels, and exchangers [2]. The SGLT5 transporter has a high affinity for fructose and a lower affinity for glucose and galactose. It is localized on the apical surface of the S2 segment of the proximal tubule, facilitating fructose reabsorption under the relevant electrochemical sodium gradient. Additionally, GLUT5, localized on the apical surface of the S3 segment, enables passive, gradient-mediated fructose transport from the tubular lumen into the tubular cells. In contrast, GLUT2, found on the basolateral surface of S2/S3 segment cells, mediates passive, gradient-driven fructose transport from the cells into the bloodstream [1,3,4].

Renal Na,K-ATPase consists of three subunits: a catalytic alpha subunit, a chaperon beta subunit, and a regulatory gamma subunit. The gamma subunit acts as a modulator when co-expressed with the alpha and beta subunits, reducing Na,K-ATPase’s affinity for sodium and potassium [5,6]. Several isoforms of subunit alpha and beta have been identified, and their functionally distinct expression in specific tissues may have different metabolic consequences.

FXYD2 (gamma subunit) was the first FXYD family protein associated with Na,K-ATPase. It is encoded by the *FXYD2* gene, which is highly expressed in the kidney, both in the medulla and cortex. The gamma subunit primarily functions to reduce Na,K-ATPase activity by decreasing its affinity for sodium and lowering its maximum velocity. This decrease in pump activity affects ion transport and membrane potential. The *FXYD2* gene is located on chromosome 11q23 and encodes two alternative splice variants, FXYD2a and FXYD2b, which differ only in their N-terminal amino acids (MTGLSMDG → MDRWYL). However, these variants have distinct distributions within the nephron and exert different modulatory effects on Na,K-ATPase activity. FXYD2a (66 amino acids) is predominantly expressed in the proximal convoluted tubule, whereas FXYD2b (64 amino acids) is found in the distal convoluted tubule (DCT), distal collecting duct, and the thick ascending limb of Henle’s loop [5,6]. Arystarkhova et al. observed the exclusive expression of the FXYD2a subunit in the proximal convoluted tubule, where over 70% of the filtered sodium is reabsorbed [5].

To date, molecular defects in the *FXYD2* gene have been described as being associated with only a single phenotype—renal isolated hypomagnesemia 2 (HOMG2; #154020). HOMG2 is a very rare disease. So far, only three families (29 patients, all descendants of a common founder) have been reported. It is an autosomal dominant disorder caused by an impairment in the function of the FXYD2b, leading to magnesium transport dysfunction in the distal convoluted tubule and resulting in primary renal magnesium wasting. Plasma levels of other electrolytes remain normal. In addition to low magnesium levels, decreased renal calcium excretion leading to hypo-calciuria is observed. Autosomal dominant primary hypomagnesemia with hypo-calciuria can be detected in childhood or in adult age. Most affected individuals are asymptomatic; however, some patients may experience generalized convulsions, indicating incomplete penetrance of *FXYD2*. In adulthood, chondrocalcinosis may develop.

In this study, we report a novel molecular variant in the *FXYD2* gene, associated with proximal tubulopathy, characterized by defective renal reabsorption of glucose, galactose, and fructose. The missense substitution c.80G>A, p.(Arg27His) is the second *FXYD2* pathogenic variant reported to date. Our findings provide the first evidence that an *FXYD2* defect (splice form a) may result in functional impairment of proximal tubular hexoses reabsorption. This novel “mutation” in the gene encoding the Na,K-ATPase gamma subunit appears to be implicated in a probable new human disease, leading to volume-dependent tubular transport disorders. This effect is likely due to the toxic impact of fructose on the nephron [7], which results in ET-1 overproduction [8] and local production of an atypical protein.

The aim of this study was to identify the potential genetic background and provide a detailed biochemical characterization of the novel proximal tubulopathy phenotype in one family (two sisters with their mother) by:

1. Evaluating 24-h urinary excretion of calcium, citrate, protein, albumin, fructose, galactose, and glucose.
2. Assessing the impact of gradually increasing volemia during a water-loading test on renal excretion of calcium, phosphate, magnesium, uric acid, citrate, glucose, and protein.
3. Investigating the effect of gradually increasing volemia in a water-loading test on renal excretion of calcium, citrate, endothelin-1 (ET-1), and protein in two sisters, with comparison to a control group.
4. Assessing urine acidification ability and tubular citrate transport in a shortened ammonium chloride (NH_4_Cl) loading test.
5. Evaluating the tubular capacity for H^+^ secretion in an oral sodium bicarbonate (NaHCO_3_) loading test, preceded by the oral administration of 300 mL/m^2^ of water.
6. Identifying the genetic cause of the observed unusual metabolic consequences.

## 2. Material and Methods

### 2.1. Patients

Two girls of Polish origin, aged 6 and 8 years (patient 1 and patient 2), were admitted to our hospital with an initial diagnosis of tubulopathy. They presented with hypercalciuria, glucosuria, and mild proteinuria. High HbA_1c_ (7.6% and 7.1%) was associated with normal glycemia, while glucose, fructose, and galactose were detected in the urine by paper chromatography. Diabetes, galactosemia, and fructosemia were excluded. The presence of these three sugars in the urine may suggest a defect in the reabsorption of fructose, galactose, and glucose in the proximal tubule. The tubular transport of phosphate, magnesium, bicarbonate, uric acid, low-molecular-weight proteins (such as α-2-microglobulin), and albumin was not impaired. Hyperaminoaciduria was confirmed in the metabolic test. High variability in the water-dependent calcium/creatinine ratio in sequential fasting urine samples, in the absence of nephrolithiasis or nephrocalcinosis, may indicate a volume-dependent tubular dysfunction in calcium, citrate, and H^+^ transport, with atypical proteinuria that does not modulate the serum concentration of electrolytes, proteins, and lipids.

### 2.2. Control Group

The age-matched control group consisted of eight children with absorptive hypercalciuria, who had no renal deposits and normal glomerular filtration rates.

### 2.3. Biochemical Analysis

Tubular dysfunction, revealed through the preliminary diagnosis in two sisters, led to the hypothesis that an increased oral water load may highlight the intensity and pattern of these defects.

The original water-loading test, which evaluated the impact of gradually increased volemia on renal excretion of several biochemical markers, involved measuring selected renal parameters before and after an oral water load (lasting 5–10 min) of 300 and 500 mL/m^2^, with an interval of one hour. The urinary ET-1 excretion was measured as previously described [9]. The diagnostic utility of the NaHCO_3_ (Chempur, Piekary Śląskie, Poland) loading test for evaluating H^+^ secretion capacity and H^+^ excretion in a short urine acidification test after NH_4_Cl (Chempur, Piekary Śląskie, Poland) loading was assessed as previously described [10]. These tests were supplemented by the measurement of citrate concentration in serum and its urine excretion.

Serum and urine electrolytes and creatinine concentrations were measured using a standard automatic chemical analyzer, while serum and urine uric acid levels were determined by an automated colorimetric procedure with a uricase–peroxidase system. Serum and urine glucose, galactose, and citrate concentrations were determined by specific enzymatic methods. Serum and urine fructose concentrations were calculated using the anthrone test, where the sum of fructose, glucose, and galactose was measured with a colorimetric method [11,12]. Aminoaciduria in the metabolic test was evaluated using a semi-quantitative method with paper chromatography.

### 2.4. Genetic Analysis

Genetic testing for all individuals was performed using genomic DNA samples automatically extracted from the peripheral blood leukocytes with a MagCore Nucleic Acid Extractor HF16Plus (RBC Bioscience, New Taipei City, Taiwan), according to the manufacturer’s protocol.

Prior to library preparation, DNA samples were quantified using the Qubit dsDNA HS Assay Kit (Life Technologies, Eugene, OR, USA), and DNA degradation was assessed by 1% agarose gel electrophoresis. About 50 ng of high-quality genomic DNA was used for library construction using the Twist Human Core Exome + Human RefSeq Panel kit (Twist Bioscience, San Francisco, CA, USA) to cover 99% of the protein-coding genes. The library was then sequenced on the NovaSeq 6000 system (Illumina, San Diego, CA, USA) according to the manufacturer’s protocol. The average read depth was 260, with >95% of the target regions covered at a depth of 20-fold. Raw FASTQ reads were mapped to the human genome assembly GRCh38/hg38. Variant calling was performed using multiple open-access algorithms: GATK HaplotypeCaller, MuTect2, FreeBayes, and DeepVariant to improve sensitivity for SNV and small indel detection. Copy number variations (CNV) were analyzed using CNVkit and Decon. Alignments were visualized with the Integrative Genomics Viewer [13]. Variant consequence annotation was performed using VEP, and the changes were further annotated with multiple data repositories, including: (1) frequency databases, such as the Genome Aggregation Database (gnomAD v4.1.0) and an in-house database specific to the Polish population, comprising data from more than 12,000 individuals suspected of having a rare genetic disease (POLdb); (2) predicted impact on protein structure and function, assessed with in silico tools, including machine learning meta-scores (BayesDel, REVEL) and individual predictors (AlphaMissense, CADD, EIGEN, FATHMM-MKL, MutationTaster, PolyPhen-2, SIFT); variants occurring at splice sites. analyzed using SpliceAI, ADA, MaxEntScan, Pangolin, and RF to assess their potential impact on normal splicing; (3) occurrence in different reference databases, such as ClinVar, the Leiden Open Variation Database (LOVD), and the Human Gene Mutation Database (HGMD) [13].

Finally, variants were interpreted based on the guidelines of the American College of Medical Genetics and Genomics and the Association for Molecular Pathology (ACMG/AMP), as well as the Association for Clinical Genomic Science (ACGS) [13,14]. According to these criteria, benign and likely benign variants were filtered out, leaving only pathogenic, likely pathogenic changes and variants of uncertain significance (VUS). Variants considered (potentially) disease-causing were correlated with the phenotype and validated in probands and segregated in family members using bi-directional Sanger sequencing with the BigDye Terminator v3.1 Kit (Applied Biosystems, Waltham, MA, USA) on the ABI 3130 Genetic Analyzer (Applied Biosystems, Waltham, MA, USA), according to the manufacturer’s protocol. The nomenclature of reported molecular variants follows the Human Genome Variation Society (HGVS) guidelines using the MANE Select human *FXYD2* reference sequence: NM_001680.5 (for cDNA) and NP_001671.2 (for protein–splice form a). Written informed consent for studies performed in the patients and their parents was obtained.

## 3. Results

### 3.1. Results of Functional Tests

The values of 24-h urinary excretion of calcium, atypical protein, albumin, citrate, glucose, galactose, and fructose in both patients and their mother are presented in Table 1. The tubular defect in reabsorption was primarily expressed for fructose, which was excreted in the urine at much higher levels than other sugars. A similar result (though in milder form) was observed in the patient’s mother.

Water load increased urinary excretion of calcium, citrate, glucose, and protein, while it did not affect the remaining parameters. Serum sodium concentration was in the lower normal range and mildly decreased after the administration of water in the loading test (Table 2).

**Figure 1:**
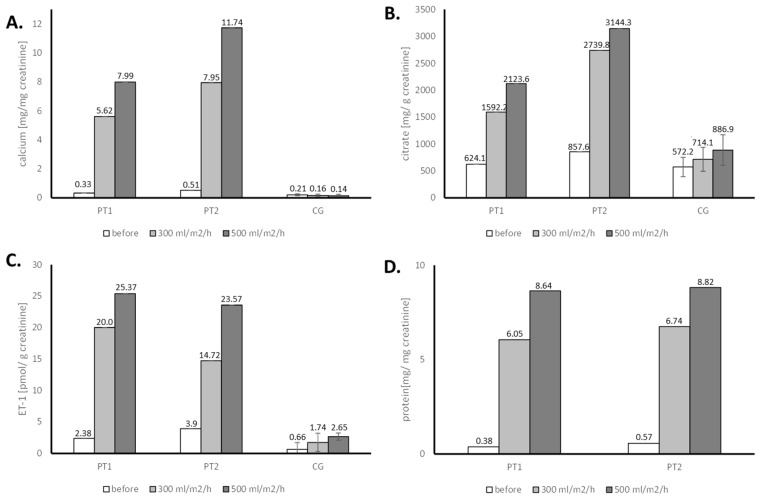
Evaluation of the urine calcium (**A**), citrate (**B**), endothelin-1 (**C**), and protein (**D**) in water loading tests for patient 1 (PT1), patient 2 (PT2) and control group (CG)-results of a single test.

In comparison to the patients, the control group showed no significant change in calciuria after water loading, with only a slight increase in citrate and ET-1 excretion (Figure 1). Statistical analysis of these results was not performed, and in any case the values presented by the patients were several times higher than in a control group.

In both patients, five hours after NH4Cl loading, a very low urine pH, accompanied by increased H^+^ excretion (as TA and NH_4_^+^), was observed, which might indicate an increased secretory capacity for H^+^. After a transient decrease in serum HCO_3_^−^, a marked increase in urinary excretion of bicarbonate and citrate was noticed, probably reflecting their low proximal reabsorption due to strong stimulation of proton pumps by systemic acidification with NH_4_Cl and the administered water (Table 3).

**Figure 2:**
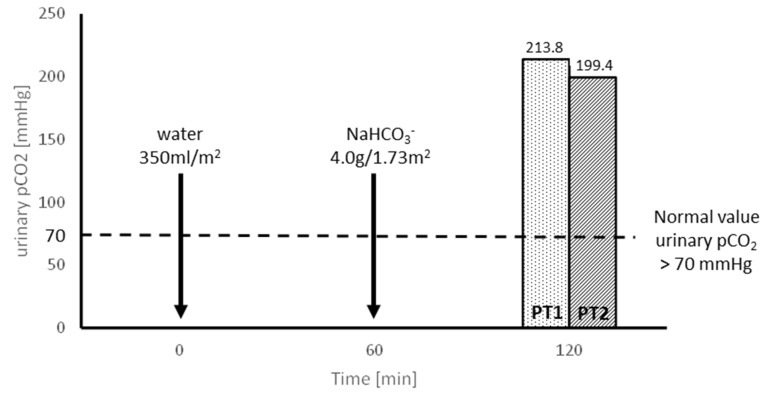
Evaluation of H^+^ secretion capacity in NaHCO_3_ loading tests, preceded by water administration, in patients 1 and 2 (PT1 and PT2).

The values of urinary carbon dioxide partial pressure (pCO_2_) after loading with NaHCO_3_ were approximately three times higher than the lower normal range. Healthy individuals should be able to increase urinary pCO_2_ to >70 mmHg and the U-B pCO_2_ gradient should be greater than 20 mmHg [15] (Figure 2).

The overall renal transport disorder suspected in the presented patients may suggest a primary defect of the SGLT5 transporter in the proximal tubule, with a secondary impact of excessive ET-1 production on nephron function.

**Table 1:** Urinary excretion of calcium, protein, albumin, citrate, glucose, galactose, and fructose for patients 1 and 2 and their mother.

Parameter	Patient 1	Patient 2	Patients Mother
Calcium mg/kg/24 h * mg/24 h	12.1	9.6	211.7 ^x^
Protein mg/24 h	1771	2392	149
Microalbuminuria mg/24 h	6.3	3.4	8.6
Citrate mg/g creat ** mg/kg/24 h *** mg/24 h	967.7 18.3	907.1 18.3	299.5 ^xx^
Glucose mg/24 h	1230.0	2040.0	26
Galactose mg/24 h	144.8	134.4	40
Fructose mg/24 h	5765.0	3979.5	715.1

Normal values in children: * <4 mg/kg/24 h; ** >400 mg/g creat; *** 8.3 ± 2.4 mg/kg/24 h; Normal values in adult females: ^x^ <250 mg/24 h; ^xx^ >320 mg/24 h [10,16].

**Table 2:** Renal excretion of calcium, magnesium, phosphate, citrate, uric acid, glucose, and protein in water loading test for patient 1 and patient 2.

Parameter	Patient 1			Patient 2		
	Before Loading	After Loading		Before Loading	After Loading	
		300 mL/m^2^	500 mL/m^2^		300 mL/m^2^	500 mL/m^2^
**Serum**						
Creatinine µmol/L	53.0		44.2	53.0		53.0
Uric acid µmol/L	261.8		261.8	202.3		202.3
Na mmol/L	137		135	137		133
Ca mmol/L	2.39		2.37	2.28		2.40
P mmol/L	1.57		1.56	1.60		1.65
Mg mmol/L	0.93		0.88	0.86		0.82
Citrate mmol/L	0.15		0.19	0.07		0.11
**Urine**						
Volume mL/min	0.52	3.83	4.10	0.57	4.50	6.00
Osmolality mOsm/kg	461	130	173	418	201	257
Ca/creat mg/mg	0.45	2.19	5.10	0.31	2.73	5.38
Mg/creat mg/mg	0.08	0.11	0.19	0.07	0.09	0.12
FE _Mg_ %	2.0	3.1	4.5	2.1	2.7	3.4
P/creat mg/mg	0.31	0.30	0.40	0.48	0.49	0.39
TRP%	96.0	96.3	95.9	94.2	94.1	95.4
TmP/GFR mmol/L	1.51	1.50	1.50	1.51	1.53	1.58
FE _uric acid_ %	4.3	6.4	6.1	6.8	7.7	8.0
Citrate/creat mg/g	1012.3	2950.6	6061.9	682.3	3099.0	5806.6
FE citrate%	21.3	58.2	106.3	30.8	108.7	166.3
Glucose/creat mg/mg	6.8	36.2	78.7	4.5	43.3	87.5
Protein/creat mg/mg	0	6.47	11.1	0.93	3.88	4.81
FE _Na_%	0.44	0.67	0.70	0.97	1.04	0.60

**Table 3:** The results of the urine acidification ability in an abbreviated ammonium chloride test for patients 1 and 2.

Parameter	Patient 1			Patient 2		
	Before Loading	After Loading		Before Loading	After Loading	
		4 h	5 h		4 h	5 h
**Serum**						
Creatinine µmol/L	44.2			35.4		
Citrate mmol/L	0.15		0.13	0.12		0.11
Plasma HCO_3_ mmol/L	24.6	19.4	20.0	24.7	19.6	21.3
**Urine**						
pH	7.12	5.02	4.49	6.99	4.74	4.56
TA μEq/min/1.73 m^2^	9.8	15.8	33.1	16.7	18.2	32.8
NH_4_ μEq/min/1.73 m^2^	8.7	40.5	43.4	22.5	53.6	47.2
HCO_3_ μEq/min/1.73 m^2^	16.8	29.3	54.6	52.4	19.1	68.4
Net H^+^ μEq/min/1.73 m^2^	−1.7	27.0	21.9	−13.2	52.7	11.6
Citrate/Creatinine mg/g	1202.0	704.8	3057.0	625.8	347.3	2117.9
FE citrate%	21.1	14.3	62.2	10.8	6.6	40.1
FE HCO_3_%	0.6	1.6	2.1	1.5	0.8	2.5

TA—titratable acid; NH_4_^+^—ammonium; HCO_3_^−^—bicarbonate; net H^+^ = TA + NH_4_^+^ −HCO_3_^−^; Normal values of short urine acidification test: pH < 5.5; NH_4_^+^ excretion > 40 μEq/min/1.73 m^2^ [15].

### 3.2. Molecular Results

**Figure 3:**
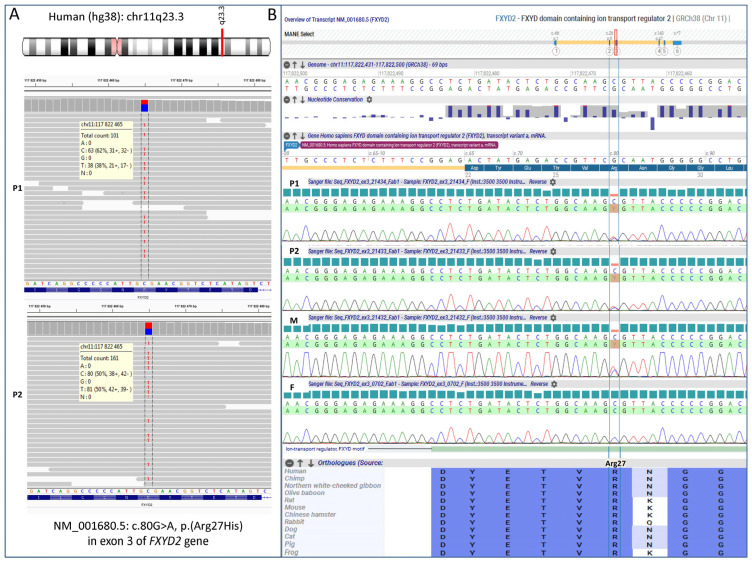
*FXYD2* genotyping results obtained by whole exome sequencing in the probands (**A**) and validated by Sanger sequencing in the entire family (**B**). P1—patient 1, P2—patient 2, M—mother, F—father.

WES analysis initially targeted genes related to renal tubulopathies (items selected by PanelApp v.4.18), particularly those involved in hexose metabolism. It then focused on evaluating variants shared by both affected sisters, revealing the presence of a heterozygous single nucleotide variant (SNV), c.80G>A, in exon 3 of the *FXYD2* gene (Figure 3A). Sanger sequencing confirmed that this variant was inherited from the mildly symptomatic mother and was absent in the healthy father (Figure 3B). The variant *FXYD2*(NM_001680.5):c.80G>A, p.(Arg27His) was found at a frequency of 0.0000163 in 1,409,560 control chromosomes in the GnomAD database, with no homozygous occurrence. It was absent in an in-house database, indicating an ultra-rare frequency. This SNV was not reported in the ClinVar, HGMD and LOVD database, but was annotated in dbSNP with rs1314602133. The variant was predicted to be damaging by 7 different pathogenicity algorithms, including, BayesDel, Alpha Missense, CADD, SIFT, FATHMM-MKL, MutationTaster, PolyPhen-2 and as ‘Uncertain’ by REVEL, EIGEN. The arginine at 27 position is weakly conserved nucleotide (phyloP100way  =  3.24, GERP RS score = 5.1), and the p.(Arg27His) variant affects the conserved Arg27 residue located in ion-transport regulator domain, between FXYD motif and transmembrane region. According ACMG/AMP system this variant has been finally classified as likely pathogenic (7 points) by in silico prediction.

Additionally, whole exome analysis excluded pathogenic/likely pathogenic variants in other genes, encoding SGLT and GLUT family proteins, including *SLC2A2* (GLUT2), *SLC2A5* (GLUT5), *SLC5A1* (SGLT1), *SLC5A2* (SGLT2), *SLC5A9* (SGLT4) and *SLC5A10* (SGLT5), in our patients.

## 4. Discussion

Several members of the FXYD family have been linked to major human diseases, including heart failure (FXYD1), hypomagnesemia (FXYD2), cancer (FXYD3, FXYD5), and schizophrenia (FXYD6), making them attractive specific targets for future therapies. To date, 27 different molecular variants (excluding benign and likely benign ones) in the *FXYD2* gene have been submitted to the ClinVar database; however, most of them (*n* = 26) have an uncertain significance (VUS) status. Only one pathogenic variant, c.121G>A p.(Gly41Arg), has been described in the *FXYD2* gene (splice form b) and is linked to autosomal dominant renal hypomagnesemia, associated with hypo-calciuria and hypokalemia [17]. It was demonstrated that artificial glycosylation (added to FXYD2 to monitor the fate of the protein in cell) of wild-type FXYD2 is reduced when co-expressed with FXYD2-Gly41Arg. These data indicate that binding of FXYD2-Gly41Arg to the wild-type FXYD2 subunit might abrogate the routing of wild-type FXYD2 to the plasma membrane, thus causing the dominant nature of this alteration [18].

Here, we report a novel, likely pathogenic variant, c.80G>A, p.(Arg27His), in the *FXYD2* gene, which correlates with a previously unreported phenotype. Family results confirm the segregation of the variant with clinical symptoms in all affected individuals. The trait of inheritance is autosomal dominant, with a 50% risk for offspring, though the penetrance and long-term effects of the disease in later life are difficult to predict. The heterozygous mother with mild symptoms presented here suggests that *FXYD2* penetrance is most likely incomplete. Expected intrafamilial variability in clinical manifestations highlights the need for further follow-up, as additional patients and families with *FXYD2*-dependent tubulopathy are reported.

Arginine is a positively charged amino acid known for its role in protein structure and function. It is often involved in forming salt bridges (ionic bonds) with negatively charged amino acids, helping to stabilize protein structures and interactions. Arginine’s guanidinium group, with its unique charge distribution and hydrogen bonding capabilities, allows it to interact effectively with negatively charged groups, like phosphates and carboxylates. Arginines are frequently found at protein–protein interfaces, playing a crucial role in stabilizing protein complexes. The gamma subunit of Na,K-ATPase (splice form a) contains six arginines, with Arg27 located after the FXYD motif near the extracellular membrane-water interface. All arginine residues may interact appreciably with the membrane, as their backbone amide sites exhibit ^1^H/^15^N HSQC signals with protection from Mn^2+^ PRE broadening. Several arginine side chains are immobilized, possibly due to hydrogen-bonded interactions with the phospholipid polar head groups. Such interactions could contribute to the membrane surface electrostatics and assist in the recruitment of specific negatively charged phospholipids, which are important for Na,K-ATPase stability and function [17,19,20]. Arginine 27 is located outside the membrane and in close proximity to the FXYD motif, therefore it is probably involved in binding to the alpha and beta subunits of ATPase. Structural modeling suggests that arginine is crucial for stabilizing the structure or interactions of the ATPase subunits and its substitution by His (which is a smaller amino acid with less positive charge and has pH-dependent properties) may alter the local conformational properties and introduce dynamic changes in the interactions of individual ATPase subunits. Since it is predicted that Arg27 may be involved in maintaining the structure of the outer loop or the interaction surface with the neighboring phospholipids, its loss or replacement with another amino acid may change the local arrangement of the helices, weaken the stability of the Na^+^/K^+^-ATPase complex or disrupt the adhesion of the protein to the cell membrane/affect its degradation. It is suggested that even subtle changes in this subunit may have clinical significance.

In this paper, we provide evidence for the molecular mechanism underlying the dominant nature of the new metabolic disorder. For the first time, we present the secondary dysfunction of the SGLT5 transporter, caused by a molecular variant in *FXYD2*, which encodes the gamma subunit of Na,K-ATPase. This variant is expressed primarily in kidney tissue (medulla and cortex) and is localized in the proximal tubules, which are responsible for the sodium-dependent reabsorption of glucose, galactose, and fructose [1,21]. Both sisters exhibited an identical defect in the transport of fructose, galactose, and glucose. Urinary excretion of fructose was much higher than that of glucose and galactose. Similar results, albeit in a milder form, were observed in the patients’ mother (Table 1). The milder phenotype in the mother was likely associated with the evaluation performed under “normal” hydration. Water load in the patients significantly increased the abnormalities, whereas water load was not used for her.

Chen et al. demonstrated a direct link between hyper-hexosemia, ET-1 activation, and subsequent functional and structural alterations in the kidneys of diabetic and galactose-fed rats [22]. Kizhner and Werman showed that long-term fructose intake in rats led to elevated urinary fructose excretion and marked proteinuria [12]. In physiology, ET-1 regulates nephron function to maintain fluid homeostasis by promoting natriuresis and antagonizing the effects of ADH in the distal nephron. ET-1 directly inhibits sodium and water reabsorption in the collecting duct, acting as an autocrine natriuretic and diuretic factor [23,24,25,26]. Therefore, the increase in ET-1 excretion in the urine collection during a water loading test in a control group is limited and reflects the physiological excretion of administered water. In contrast, ET-1 excretion in our patients, under hypervolemic conditions, is several times higher as when stimulated by hexoses [22].

Water load in the two sisters resulted in increased diuresis, along with a parallel increase in urine ET-1, citrate, calcium, and atypical protein excretion (Figure 1). ET-1 overproduction stimulates proton pumps, leading to increased secretion of H^+^ in the distal nephron [27]. This was confirmed in both sisters during the loading test with NaHCO3, preceded by the water load (Figure 2). The increased H^+^ secretion is associated with equivalent bicarbonate production, which is transported to the blood. This secondary effect leads to a decrease in proximal citrate reabsorption, ultimately resulting in hypercitraturia. The increase in calciuria and citraturia after the water load was induced by transient hypervolemia, which caused a secondary decrease in the reabsorption of water, sodium, and calcium with citrate in the proximal tubule. The genetic variation in the ATPase gamma subunit, which likely disrupts sodium-dependent hexose reabsorption in the proximal tubule and might lead to functional decreases in sodium reabsorption under increased volume conditions, resulted in glucosuria during the water loading test (Table 2). In the presence of the mutated gamma subunit on the basolateral surface of proximal tubule cells, secondary dysfunctions of sodium-dependent transporters for hexoses and amino acids may occur. We conclude that fructose transport in the proximal tubule occurs primarily through Na^+^-linked co-transport processes. In normal individuals, a fructose-enriched diet enhances the reabsorption and metabolism of fructose in the proximal tubule [28]. The transport of fructose across the luminal and basolateral cell membranes is mediated by SGLT5 and GLUT2, respectively. Therefore, a genetically driven defect in these transporters leads to the disturbances observed in our patients.

**Figure 4:**
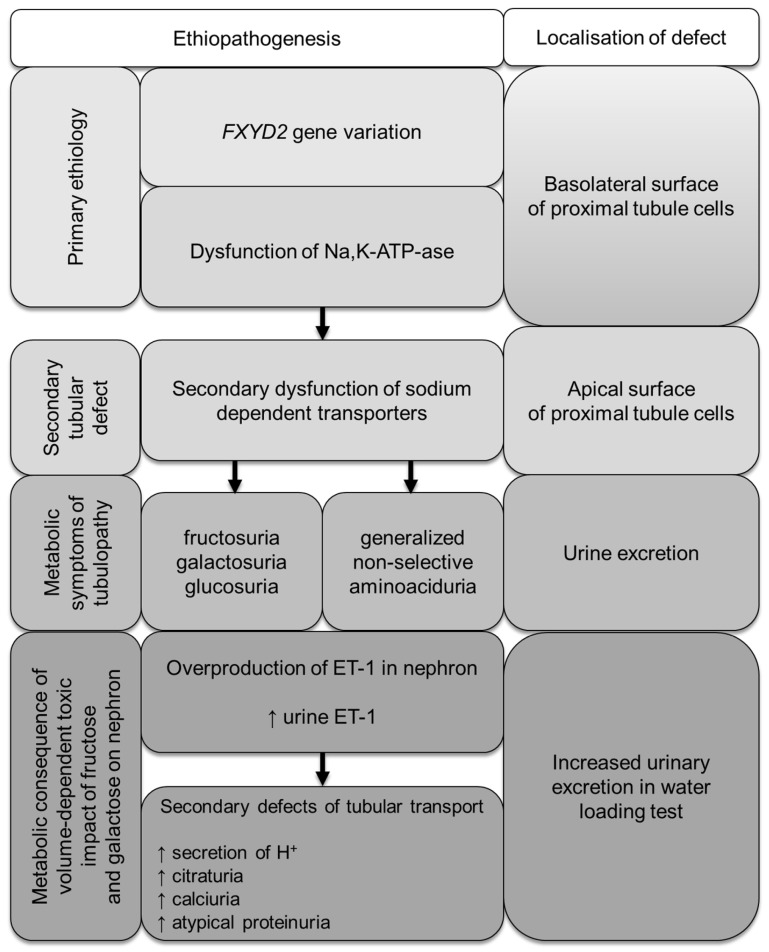
Metabolic consequences of *FXYD2* gene variation in patients with familial renal hexosuria.

Our experimental evidence suggests the functional role of FXYD proteins in the regulation of Na,K-ATPase in a tissue- and splice form-specific manner. Both splice variants, FXYD2a and FXYD2b, produce identical effects on the catalytic and transport properties of Na,K-ATPase. However, the functional effects of FXYD2a and FXYD2b differ, likely due to unidentified post-translational modifications that may be cell-specific or dependent on the physiological state. For example, FXYD2a is mainly found in PCT, while FXYD2b is more abundant in DCT. The defect in glucose, galactose, and fructose renal reabsorption in the proximal tubule, as described above, extends the phenotypic spectrum associated with Na,K-ATPase dysfunction. It is linked to the molecular change in the *FXYD2* gene leading to dysregulation of the FXYD2a splice isoform (as summarized in Figure 4). In patients with isolated dominant hypomagnesemia, the FXYD2b abnormality causes inadequate Na,K-ATPase-energized transcellular magnesium transport in the distal convoluted tubule via the apical Mg^2+^ channel TRPM6 and the Na^+^-Mg^2+^ exchanger at the basolateral site [18,29]. In our patients, we present the secondary dysfunction of the SGLT transporter, caused by a molecular variant in FXYD2a, which is expressed primarily in kidney tissue (medulla and cortex) and is localized in the proximal tubules. This providing further support for different functional roles of *FXYD2* variants.

## 5. Conclusions

Molecular analysis conducted in both patients and their mother led to the final diagnosis of primary disease: familial proximal tubulopathy. This analysis also identified the underlying complex mechanisms regulating renal homeostasis and their clinical relevance. In both sisters, the molecular defect in the Na,K-ATPase transport system likely impaired the generation of the appropriate Na^+^ and K^+^ gradients across the basolateral membrane of the proximal tubule, which are essential for activating sodium-dependent tubular reabsorption of hexoses. This dysfunction resulted in renal loss of fructose, galactose, and glucose. Our study provides functional evidence that molecular variants in the *FXYD2* gene lead to Na,K-ATPase dysfunction and supports the association of *FXYD2* with a new disease: sodium-related familial renal hexosuria. A limitation of this report is that it is based on data from only one family. Therefore, the proposed patho-mechanism of FXYD2-related metabolic disease needs to be verified in other patients with similar biochemical defects. It is also important to note that some underdiagnosed cases may exhibit incomplete penetrance or mild symptoms, as observed in the HOMOG2 phenotype and in the mother of the presented patients. For this reason, all individual cases with atypical nephropathy should undergo a thorough diagnostic workup, following a detailed diagnostic algorithm.

## Data Availability

The original contributions presented in this study are included in the article. Further inquiries can be directed to the corresponding author.

## References

[1] Gonzalez-Vincente A., Cabral P., Hong N., Asirwatham J., Saez F., Garvin J. (2019). Fructose reabsorption by rat proximal tubules of Na^+^-linked cotransporters and the effect of dietary glucose. Am. J. Physiol. Ren. Physiol..

[2] Feraille E., Doucet A. (2001). Sodium-potassium-adenosinetriphosphatase-dependent sodium transport in the kidney hormnal control. Physiol. Rev..

[3] Sugawara-Yokoo M., Suzuki Y., Matsuzaki T., Naruse T., Takata K. (1999). Presence of fructose transporter GLUT5 in the S3 proximal tubules in the rat kidney. Kidney Int..

[4] Douard V., Ferraris R. (2008). Regulation of the fructose transporter GLUT5 in health and disease. Am. J. Physiol. Endocrinol. Metab..

[5] Arystarkhova E., Wetzel R.K., Sweadner K.J. (2002). Distribution and oligomeric association of splice forms of Na(+)-K(+)-ATPase regulatory γ-subunit in rat kidney. Am. J. Physiol. Ren. Physiol..

[6] Arystarkhova E., Donnet C., Asinovski N.K., Sweadner K.J. (2002). Differential regulation of renal Na,K-ATPase by splice variants of the γ subunit. J. Biol. Chem..

[7] Aoyama M., Isshiki K., Kume S., Chin-Kanasaki M., Araki H., ArakiSI Koya D., Haneda M., Kashiwagi A., Maegawa H., Uzu T. (2012). Fructose induces tubulointerstitial injury in the kidney of mice. Biochem. Biophys. Res. Commun..

[8] Marsen T.A., Schramek H., Dunn M.J. (1994). Renal actions of endothelin: Linking cellular signaling pathways to kidney disease. Kidney Int..

[9] Grenda R., Wühl E., Litwin M., Janas R., Sladowska J., Arbeiter K., Berg U., Caldas-Afonso A., Fischbach M., Mehls O. (2007). Urinary excretion of endothelin-1 (ET-1), transforming growth factor-beta1 (TGF-beta1) and vascular endothelial growth factor (VEGF165) in paediatric chronic kidney diseases: Results of the ESCAPE trial. Dial. Transplant..

[10] Zawadzki J. (1998). Permeability defect with bicarbonate leak as a mechanism of immune-related distal renal tubular acidosis. Am. J. Kidney Dis..

[11] Roe H.G. (1934). A colorimetric methods for the determination of fructose in blood and urine. J. Biol. Chem..

[12] Kizhner T., Werman M. (2002). Long-term fructose intake: Biochemical consequences and altered renal histology in the male rat. Metabolism.

[13] Halat-Wolska P., Ciara E., Pac M., Obrycki Ł., Wicher D., Iwanicka-Pronicka K., Bielska E., Chałupczyńska B., Siestrzykowska D., Kostrzewa G. (2025). Molecular Review of Suspected Alport Syndrome Patients-A Single-Centre Experience. Genes.

[14] Richards S., Aziz N., Bale S., Bick D., Das S., Gastier-Foster J., Grody W.W., Hegde M., Lyon E., Spector E. (2015). Standards and guidelines for the interpretation of sequence variants: A joint consensus recommendation of the American College of Medical Genetics and Genomics and the Association for Molecular Pathology. Genet. Med..

[15] Santos F., Ordóñez F.A., Claramunt-Taberner D., Gil-Peña H. (2015). Clinical and laboratory approaches in the diagnosis of renal tubular acidosis. Pediatr. Nephrol..

[16] Srivastava T., Alon U.S. (2007). Pathophysiology of hypercalciuria in children. Pediatr. Nephrol..

[17] de Baaij J.H., Dorresteijn E.M., Hennekam E.A., Kamsteeg E.J., Meijer R., Dahan K., Muller M., van den Dorpel M.A., Bindels R.J., Hoenderop J.G. (2015). Recurrent FXYD2 p.Gly41Arg mutation in patients with isolated dominant hypomagnesaemia. Nephrol. Dial. Transplant..

[18] Cairo E.R., Friedrich T., Swarts H.G., Knoers N.V., Bindels R.J., Monnens L.A., Willems P.H., De Pont J.J., Koenderink J.B. (2008). Impaired routing of wild type FXYD2 after oligomerisation with FXYD2-G41R might explain the dominant nature of renal hypomagnesemia. Biochim. Biophys. Acta.

[19] Arystarkhova E., Sweadner K.J. (2016). Functional Studies of Na(+),K(+)-ATPase Using Transfected Cell Cultures. Methods Mol. Biol..

[20] Gong X.M., Ding Y., Yu J., Yao Y., Marassi F.M. (2015). Structure of the Na,K-ATPase regulatory protein FXYD2b in micelles: Implications for membrane-water interfacial arginines. Biochim. Biophys. Acta.

[21] Grempler R., Augustin R., Froehner S., Hildebrandt TSimon E., Mark M., Eickelmann P. (2012). Functional characterization of human SGLT-5 as a novel kidney-specific sodium-dependent sugar transport. FEBS Lett..

[22] Chen S., Evans T., Deng D., Cukiernik M., Chakrabarti S. (2002). Hyperhexosemia induced functional and structural changes in the kidneys: Role of endothelins. Nephron.

[23] Gastone G., Serneri N., Modesti P., Cecioni I., Biagini D., Costoli A., Colella A., Naldoni A., Paoletti P. (1995). Plasma endothelin and renal endothelin are two distinct systems involved in volume homeostasis. Am. J. Physiol. Heart Circ. Physiol..

[24] Matthyus I., Zimmerhackl L., Schwartz A., Hentschel M., Brandis M., Miltenyi M., Tulassay T. (1994). Renal excretion of endothelin in children is influenced by age and diuresis. Acta Paediatr..

[25] Kohan D., Padilla E. (1993). Osmolar regulation of endothelin-1 production by rat inner medullary collecting duct. J. Clin. Investig..

[26] Pandit M., Inscho E., Zhang S., Seki T., Rohatgi R., Gusella L., Kishore B., Kohan D. (2015). Flow regulation of endothelin-1 production in the inner medullary collecting duct. Am. J. Physiol. Ren. Physiol..

[27] Wesson D. (1997). Endogenous endothelins mediate increased distal tubule acidification induced by dietary acid in rats. J. Am. Soc. Clin. Investig..

[28] Nakagawa T., Johnson R.J., Andres-Hernando A., Roncal-Jimenez C., Sanchez-Lozada L.G., Tolan D.R., Lanaspa M.A. (2020). Fructose production and metabolism in the kidney. J. Am. Soc. Nephrol..

[29] Viering D.H.H.M., de Baaij J.H.F., Walsh S.P., Kleta R. (2017). Genetic causes of hypomagnesemia, a clinical overview. Pediatr. Nephrol..

